# PS-05 - MEASLES SUSCEPTIBILITY IN CHILDREN AND YOUNG PEOPLE IN SCOTLAND – IDENTIFYING AREAS FOR TARGETED MMR VACCINATION CATCH-UP

**DOI:** 10.1093/pubmed/fdag046.054

**Published:** 2026-07-09

**Authors:** Dr Hunter Leonie, Ms Salomi Barkat, Mr Greg Powell, Mr Neil Perkins, Ms Adriana Zalewska, Dr Daniel Chandler, Dr Cheryl Gibbons

**Affiliations:** Public Health Scotland; Public Health Scotland; Public Health Scotland; Public Health Scotland; Public Health Scotland; Public Health Scotland; Public Health Scotland

## Abstract

**Background:**

In 2024, the UK lost its WHO measles elimination status. Although laboratory-confirmed measles remains relatively low in Scotland, cases were higher in 2025 (n=28) and 2024 (n=24) than in recent previous years. In 2025, there were two outbreaks in under-vaccinated communities. MMR coverage has been gradually declining in Scotland, and two dose MMR coverage is below the recommended WHO target of 95%. There are variations in uptake and concentrations of low coverage in Scotland associated with geographical location, deprivation and ethnicity.

**Objectives:**

To estimate measles susceptibility of pre-school and school aged cohorts in Scotland, to inform public health action. To identify geographic areas for targeted vaccination catch up activities.

**Methods:**

Measles susceptibility was calculated for pre-school, primary school and secondary school cohorts in Scotland at health board level, local authority, and intermediate zone in 2025. Inequalities in measles susceptibility were described using the Scottish Index of Multiple Deprivation (SIMD) and urban rural classification.

**Results:**

In 2025, measles susceptibility in pre-school aged children (1 to 4 years), primary school aged children and secondary school aged children was 10.3%, 9.2% and 8.6% respectively. There was variation in susceptibility between and within geographic health boards. Measles susceptibility was higher in children and young people residing in the most deprived areas (SIMD quintile 1) compared to the least deprived areas (SIMD quintile 5).

**Conclusion:**

Measles susceptibility in Scotland was below the UK target of 15% in pre-school aged children but above the recommended 5% in school aged children. Geographic analysis identified areas for prioritising targeted MMR catch up and has been shared with health boards. Increasing measles vaccination coverage in school aged children is important to prevent outbreaks, protect vulnerable individuals and re-gain measles elimination status.

